# Supplementary material for: Pharmacokinetics of Curcumin Delivered by Nanoparticles and the Relationship with Antitumor Efficacy: A Systematic Review
Source: Pharmaceuticals (Basel). 2023 Jun 29;16(7):943. doi: 10.3390/ph16070943 (PMC10384157; doi:10.3390/ph16070943)
Supplement: Supplementary file 1 [file pharmaceuticals-16-00943-s001.zip › Table S1. Reasons for exclusion from studies.pdf]

Tabela S1. Reasons for exclusion from studies

Rason	Quantity
Not published in the last 5 years	78
HPLC method not used	52
This is a Systematic Review	6
Excluded due to technical difficulties of the tools	12
Study with an animal that does not contain a tumor	36
in vitro study	7
Use of curcumin as a drug	21
Nanoparticle loaded with materials other than curcumin	4
It's not about nanostructures	2
There was no time-limit analysis	3
**Total**	221
