# Supplementary material for: Pharmacokinetics of Curcumin Delivered by Nanoparticles and the Relationship with Antitumor Efficacy: A Systematic Review
Source: Pharmaceuticals (Basel). 2023 Jun 29;16(7):943. doi: 10.3390/ph16070943 (PMC10384157; doi:10.3390/ph16070943)
Supplement: Supplementary file 1 [file pharmaceuticals-16-00943-s001.zip › Table S2 - Risk of bias included studies.pdf]

Table S2. Risk of bias of selected individual studies.

	Criteria used for publication risk analysis
**1**	Are the experimental groups comparable and the control groups available?
**2**	Is the number of experimental units in each group clearly stated? And is it the same number (n) that was evaluated in the statistical analyses?
**3**	Have pre-established criteria for inclusion or exclusion of experimental units been described during the experiments or during the analyses?
**4**	Were pre-established criteria for inclusion or exclusion of experimental units described during the experiments or during the analyses? (Applies only to in vivo studies).
**5**	Is there a description of the use of a method for blinding the researchers, especially those responsible for handling the animals and analysing the results? (Applies only to in vivo studies).
**6**	Were the biological variables used to measure the results (conclusions) clearly described?
**7**	Were the details of the statistical analysis used in each analysis provided?
**8**	Has all relevant information about the characteristics of the animals and cells been clearly described?
**9**	Are the steps of the experimental procedures and their ranges and measurements clearly described and detailed enough to allow replication?
**10**	Do the results include data from all experiments clearly described with an indication of the value of the statistically significant difference (p-value)?
**11**	Is the summary of the study clear and does it include all relevant information? Such as objectives, experimental model, key methods used and relevant results?
**12**	Does the introduction provide information that contextualises and justifies the conduct of the study?
**13**	Is the research question clearly described in the study objectives?
**14**	In vitro - Is there a description of the approval of the use of cells, in the case of human donors only, by an ethics committee and has the name of the committee been provided? If no approval was granted, is there a rationale for conducting the study?<br><br>In vivo - Is there a description of the approval for the use of animals by an ethics committee and was the name of the committee given? If there was no approval, is there a rationale for conducting the study?

15 Were the experimental conditions for the allocation and supplementation of animals clearly stated? (Applies only to in vivo studies).

16 Were procedures described to reduce stress, pain and suffering of the animals? (Applies only to in vivo studies)

17 Is the interpretation of the results related to the objectives of the study? And have the limitations of the study been described?

18 Is there a description of the results that indicates the possibility of transfer to future experiments in other experimental models or not? (For in vivo or clinical studies)

19 Is there information about a study protocol that was developed before the experiments began? If so, is there an indication of where it was published? (Applies only to in vivo studies).

20 Did the study provide raw data on the results? Note: If this item is not present, the study should not be considered at high risk of bias, but this information is not available.

21 Is there a statement about the presence or absence of conflicts of interest in the study?

ROB 2.0 platform.
