# Supplementary material for: Pharmacokinetics of Curcumin Delivered by Nanoparticles and the Relationship with Antitumor Efficacy: A Systematic Review
Source: Pharmaceuticals (Basel). 2023 Jun 29;16(7):943. doi: 10.3390/ph16070943 (PMC10384157; doi:10.3390/ph16070943)
Supplement: Supplementary file 1 [file pharmaceuticals-16-00943-s001.zip › Table S3. Database serch and results.pdf]

Table S3. Database Search and results

Base	String	Resultado	Data
Embase	'curcumin'/exp OR 'curcuma longa' AND 'pharmacokinetics'/exp AND 'nanoparticle'/exp OR 'nanocomposite'/exp OR 'nanoconjugate'/exp OR 'nanomaterial'/exp OR 'nanocrystal'/exp OR 'nanosphere'/exp OR 'nanoemulsion'/exp OR 'nanocapsule'/exp OR 'liposome'/exp AND 'malignant neoplasm'/exp OR 'neoplasm'/exp OR (neoplasm* AND benign) AND 'antineoplastic agent'/exp	3	23/09/21
ScienceDirect	(Curcumin) AND ("Drug delivery" OR pharmacokinetics) AND (Cancer OR Tumour) AND (Nanoparticle OR Nanostructure) AND (“anticancer drug”)	35	01/10/21
Scopus	((TITLE-ABS-KEY(curcumin*) OR TITLE-ABS-KEY("yellow tumeric") OR TITLE-ABS-KEY(diferuloylmethane) OR TITLE-ABS-KEY("1,7 bis (4 hydroxy 3 methoxyphenyl) 1,6 heptadiene 3,5 dione") OR TITLE-ABS-KEY("bis (4 hydroxy 3 methoxycinnamoyl) methane")OR TITLE-ABS-KEY(curcumine) OR TITLE-ABS-KEY(nanocurc) OR TITLE-ABS-KEY("curcuma longa")) AND PUBYEAR > 2010 AND PUBYEAR < 2022) AND ((TITLE-ABS-KEY(pharmacokinetics) OR TITLE-ABS-KEY("drug kinetics") OR TITLE-ABS-KEY("kinetics and drug") OR TITLE-ABS-KEY(admet) OR TITLE-ABS-KEY(adme-tox) OR TITLE-ABS-KEY("absorption and distribution") OR TITLE-ABS-KEY("metabolism and elimination and toxicology") OR TITLE-ABS-KEY(ladmer) OR TITLE-ABS-KEY("liberation and absorption and distribution and metabolism and elimination and response") OR TITLE-ABS-KEY("absorption and distribution and metabolism")) AND PUBYEAR > 2010 AND PUBYEAR < 2022) AND ((TITLE-ABS-KEY(nanoparticles) OR TITLE-ABS-KEY(nanocomposites) OR TITLE-ABS-KEY(nanoconjugates) OR TITLE-ABS-KEY("material and nanostructured") OR TITLE-ABS-KEY("materials and nanostructured") OR TITLE-ABS-KEY(nanomaterial) OR TITLE-ABS-KEY(nanostructure) OR TITLE-ABS-KEY("nanostructured material") OR TITLE-ABS-KEY("nanostructured materials") OR TITLE-ABS-KEY(nanostructure*) OR TITLE-ABS-KEY("material and nanocrystalline") OR TITLE-ABS-KEY("nanocrystalline material") OR TITLE-ABS-KEY(nanocrystals) OR TITLE-ABS-KEY(nanocrystal) OR TITLE-ABS-KEY(nanospheres) OR TITLE-ABS-KEY(nanoemulsions) OR TITLE-ABS-KEY(nanocapsules) OR TITLE-ABS-KEY(liposomes)) AND PUBYEAR > 2010 AND PUBYEAR < 2022) AND ((TITLE-ABS-KEY (cancer) OR TITLE-ABS-KEY(cancers) OR TITLE-ABS-KEY("benign neoplasm") OR TITLE-ABS-KEY("benign neoplasms") OR TITLE-ABS-KEY(malignancies) OR TITLE-ABS-KEY(malignancy) OR TITLE-ABS-KEY("malignant neoplasm") OR TITLE-ABS-KEY("malignant neoplasms") OR TITLE-ABS-KEY(neoplasia) OR TITLE-ABS-KEY(neoplasias) OR TITLE-ABS-KEY(neoplasm) OR TITLE-ABS-KEY("neoplasm and benign") OR TITLE-ABS-KEY("neoplasm and malignant") OR TITLE-ABS-KEY("neoplasms and benign") OR TITLE-ABS-KEY(tumors) OR TITLE-ABS-KEY(tumor) OR TITLE-ABS-KEY("neoplastic disease") OR TITLE-ABS-KEY("tumoral entity") OR TITLE-ABS-KEY("tumoral mass") OR TITLE-ABS-KEY("tumorous entity") OR TITLE-ABS-KEY("tumorous mass") OR TITLE-ABS-KEY(tumour) OR TITLE-ABS-KEY("tumor and malignant") OR TITLE-ABS-KEY("tumour and malignant")) AND PUBYEAR > 2010 AND PUBYEAR < 2022) AND ((TITLE-ABS-KEY(antitumor) OR TITLE-ABS-KEY("anti neoplastic agent") OR TITLE-	108	29/09/21

	ABS-KEY("anticancer agent") OR TITLE-ABS-KEY("anticancer drug") OR TITLE-ABS-KEY(anticancerogen) OR TITLE-ABS-KEY(anticarcinogen) OR TITLE-ABS-KEY("anticarcinogenic agents") OR TITLE-ABS-KEY("antineoplastic agents") OR TITLE-ABS-KEY(combined) OR TITLE-ABS-KEY(phytogenic)OR TITLE-ABS-KEY("antineoplastic combined chemotherapy protocols") OR TITLE-ABS-KEY("antitumor agent") OR TITLE-ABS-KEY("cancer chemotherapeutic agent") OR TITLE-ABS-KEY("cancer inhibitor") OR TITLE-ABS-KEY("tumor inhibitor") OR TITLE-ABS-KEY("tumour inhibitor")) AND PUBYEAR > 2010 AND PUBYEAR < 2022)		
Web of Science	1. TS=("1, 7 bis (4 hydroxy 3 methoxyphenyl) 1, 6 heptadiene 3, 5 dione" OR "bis (4 hydroxy 3 methoxycinnamoyl) methane" OR "Curcuma longa" OR curcumin* OR diferuloylmethane OR nanocurc* OR "turmeric yellow")<br>2. TS=((Absorption AND Distribution AND Metabolism AND Elimination) OR (Absorption AND Distribution AND Metabolism AND Elimination AND Toxicology) OR ADME OR ADMET OR ADME-Tox OR "body drug relation" OR "drug body relation" OR "Drug Kinetics" OR (Kinetics AND Drug) OR LADMER OR (Liberation AND Absorption AND Distribution AND Metabolism AND Elimination AND Response) OR "pharmaceutical kinetic" OR pharmacokinetic OR pharmaco-kinetic OR Pharmacokinetics OR "pharmacological kinetic")<br>3. TS=(Liposomes OR (Material* AND Nanocrystalline) OR (Material* AND Nanostructured) OR Nanocapsules OR Nanocomposite* OR Nanoconjugate* OR Nanocrystal* OR "Nanocrystalline Material*" OR Nanoemulsions OR Nanomaterial* OR Nanoparticle* OR Nanospheres OR Nanostructure* OR "Nanostructured Material*")<br>4. TS=("Benign Neoplasm*" OR Cancer* OR Malignancies OR Malignancy OR "malignant neoplasia" OR "Malignant Neoplasm*" OR "malignant neoplastic disease" OR "malignant tumor" OR "malignant tumour" OR Neoplasia* OR (neoplasia AND malignant) OR Neoplasm* OR (Neoplasm* AND Benign) OR (Neoplasm* AND Malignant) OR "neoplastic disease" OR tumor* OR (tumor AND malignant) OR "tumoral entity" OR "tumoral mass" OR "tumorous entity" OR "tumorous mass" OR tumour OR (tumour AND malignant) OR "tumoural entity" OR "tumoural mass" OR "tumourous entity" OR "tumourous mass")<br>5. TS=("anti cancer drug" OR "anti neoplastic agent" OR (Antibodies AND Neoplasm) OR (Antibodies AND Tumor) OR "anticancer agent" OR "anticancer drug" OR anticancerogen OR anticarcinogen OR "anticarcinogenic agents" OR "antineoplastic agent*" OR (antineoplastic agents AND combined) OR (antineoplastic agents AND phytogenic) OR "antineoplastic combined chemotherapy protocols" OR "antineoplastic drug" OR "antineoplastic peptide" OR antineoplastics OR (antineoplastics AND enzymes) OR (antineoplastics AND miscellaneous) OR (antineoplastics AND radiopharmaceuticals) OR (antineoplastics AND signal transduction inhibitors) OR "antitumor agent" OR "antitumor drug" OR Antitumor OR antitumoral OR "antitumour agent" OR "antitumour drug" OR "cancer chemotherapeutic agent" OR "cancer inhibitor" OR "carcinostatic drug" OR (drug AND antineoplastic) OR "Neoplasm Antibodies" OR "Tumor Antibodies" OR "tumor inhibitor" OR "tumour inhibitor")	91	30/09/21
Pubmed	(((("Curcumin"[Mesh]) AND ("Pharmacokinetics"[Mesh])) AND ("Neoplasms"[Mesh])) AND ((((("Nanoparticles"[Mesh]) OR ("Nanostructures"[Mesh])) OR ("Nanocomposites"[Mesh])) OR ("Nanoconjugates"[Mesh])) OR ("Liposomes"[Mesh]))) AND (("Antibodies, Neoplasm"[Mesh]) OR ("Antineoplastic Agents"[Mesh]))	103	29/09/21

Lilacs	(curcumina) OR (curcuma longa) OR (Amarelo Turmérico) OR (nanocápsula) OR (Diferulometano) AND ((pharmacokinetics) OR (farmacocinética) OR (distribuição tecidual) OR (toxicocinética)) AND ((Nanopartículas) OR (Materiais Nanocristalinos) OR (Nanocristais)) AND ((Câncer) OR (Neoplasias) OR (Neoplasia Benigna) OR (Neoplasia Maligna) OR (Tumor) OR (Tumores) OR (Tumor Maligno) OR (Neoplasias Malignas)) AND ((antitumor drugs) OR (Antineoplastic Agents) OR (Agente Antitumoral) OR (Fármaco Antitumoral) OR (antitumoral) OR (Agentes Quimioterápicos Anticâncer)) AND ((antitumor drugs) OR (Antineoplastic Agents) OR (Agente Antitumoral) OR (Fármaco Antitumoral) OR (antitumoral) OR (Agentes Quimioterápicos Anticâncer)) AND ((Câncer) OR (Neoplasias) OR (Neoplasia Benigna) OR (Neoplasia Maligna) OR (Tumor) OR (Tumores) OR (Tumor Maligno) OR (Neoplasias Malignas)) AND ((Nanopartículas) OR (Materiais Nanocristalinos) OR (Nanocristais)) AND ((pharmacokinetics) OR (farmacocinética) OR (distribuição tecidual) OR (toxicocinética))	0	13/09
Clinical Trials	(Neoplasms) AND (Curcumin OR “Curcuma longa”) AND (pharmacokinetics OR Kinetics)	5	23/09
Scielo	Curcumina: ((curcumin* OR “yellow tumeric” OR diferuloylmethane OR (“1,7 bis (4 hydroxy 3 methoxyphenyl) 1,6 heptadiene 3,5 dione”) OR (“bis (4 hydroxy 3 methoxycinnamoyl) methane”) OR (curcumine OR nanocurc OR “curcuma longa”)) Farmacocinética: ((pharmacokinetics OR “drug kinetics” OR (kinetics AND Drug) OR ADMET OR ADME-Tox OR (absorption AND Distribution) OR (Metabolism AND Elimination AND Toxicology) OR LADMER OR (liberation AND absorption AND distribution AND metabolism AND elimination AND response) OR (“absorption AND distribution AND metabolism)) Nanopartículas: ((nanoparticles OR nanocomposites OR nanoconjugates OR (material AND nanostructured) OR (Materials AND nanostructured) OR nanomaterial OR nanostructure OR “nanostructured material” OR “nanostructured materials” OR nanostructure* OR (Material AND Nanocrystalline) OR “Nanocrystalline Material” OR Nanocrystals OR Nanocrystal OR Nanospheres OR Nanoemulsions OR Nanocapsules OR liposomes)) Cancer: ((cancer OR cancers OR “benign neoplasm” OR “benign neoplasms” OR malignancies OR malignancy OR “malignant neoplasm” OR “malignant neoplasms” OR neoplasia OR neoplasias OR neoplasm OR (neoplasm AND benign) OR (neoplasm AND malignant) OR (neoplasms AND benign) OR tumors OR tumor OR “neoplastic disease” OR “tumoral entity” OR “tumoral mass” OR “tumorous entity” OR “tumorous mass” OR tumour OR (tumor AND malignant) OR (tumour AND malignant)) Antitumor: ((antitumor OR “anti neoplastic agent” OR “anticancer agent” OR “anticancer drug” OR anticancerogen OR anticarcinogen OR “anticarcinogenic agents” OR (“antineoplastic agents” AND combined) OR phytogenic OR	0	13/09/21

	“antineoplastic combined chemotherapy protocols” OR “antitumor agent” OR “cancer chemotherapeutic agent” OR “cancer inhibitor” OR “tumor inhibitor” OR “tumour inhibitor”))		
